# Comparative Population Genetics of Two Alvinocaridid Shrimp Species in Chemosynthetic Ecosystems of the Western Pacific

**DOI:** 10.1111/1749-4877.12954

**Published:** 2025-01-27

**Authors:** Qi Dai, Ting Xu, Yixuan Li, Yanan Sun, Yitao Lin, Takuya Yahagi, Maeva Perez, Pei‐Yuan Qian, Jian‐Wen Qiu

**Affiliations:** ^1^ Department of Biology Hong Kong Baptist University Hong Kong SAR China; ^2^ Southern Marine Science and Engineering Guangdong Laboratory (Guangzhou) Guangzhou China; ^3^ Department of Ocean Science The Hong Kong University of Science and Technology Hong Kong SAR China; ^4^ Laboratory of Marine Organism Taxonomy and Phylogeny, Qingdao Key Laboratory of Marine Biodiversity and Conservation Institute of Oceanology, Chinese Academy of Sciences Qingdao China; ^5^ Department of Marine Ecosystem Science, Atmosphere and Ocean Research Institute The University of Tokyo Kashiwa Chiba Japan

**Keywords:** Alvinocarididae, biogeography, chemosynthesis‐based ecosystem, genetic diversity, population connectivity

## Abstract

Deep‐sea shrimps from the family Alvinocarididae are prominent inhabitants of chemosynthesis‐based habitats worldwide. However, their genetic diversity and population connectivity remain poorly understood due to limited sampling. To fill these knowledge gaps, we compared the population genetics of two vent‐ and seep‐dwelling alvinocaridid species with overlapped geographic ranges between the South China Sea and the Manus Basin. *Alvinocaris longirostris* has a wider distribution, ranging from 35°N to 3°S and at depths of 930 to 1736 m, while *Alvinocaris kexueae* is more restricted, found between 16°N and 3°S at depths of 1300 to 1910 m. Our analysis, based on the mitochondrial *cytochrome c oxidase subunit 1* gene, revealed that *A. longirostris* had lower genetic diversity and minimal genetic differentiation across eight disjoint vent and seep populations. In contrast, the narrower‐distributed *A*. *kexueae* exhibited higher genetic diversity and significant genetic differentiation, with stronger gene flow observed from its Haima seep population to the Manus Basin vent population. In addition, both species appear to have experienced population expansion in their recent evolutionary history. These results suggest that *A. longirostris* and *A. kexueae* may possess distinct life‐history traits that contribute to their differing distribution ranges in the Western Pacific.

## Graphical abstract

Little is known about the population divergence and gene flow of deep‐sea animals living in disjunct hydrothermal vents and cold seep habitats. Taking advantage of samples collected from multiple cruises across a huge distance of >5000 km, we revealed the differential population divergence pattern and gene flow in two congeneric species of shrimps living in these chemosynthetic ecosystems.

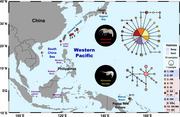

## 1 Introduction

Hydrothermal vents and hydrocarbon seeps are two types of chemosynthesis‐based ecosystems with remarkable biomass. Vent fields are typically found in active tectonic areas, such as mid‐ocean ridges or in back‐arc spreading centers, while seep areas usually occur on both active and passive continental margins (Levin et al. 2016; Suess 2014; Van Dover et al. 2002). Both habitats are considered ephemeral and patchy, although vent fields tend to be more ephemeral than seep areas. These habitats are sustained by geofluids in the deep ocean, which are influenced by geological activities, and are often separated by considerable distances of up to thousands of kilometers (Levin et al. 2016; Van Dover et al. 2002). Like shallow‐water macrobenthos, most adult deep‐sea macrobenthos are either sessile or exhibit limited motility. Their populations achieve connectivity among isolated vent and seep habitats primarily through a pelagic phase during their early life stages (Arellano et al. 2014; Pineda, Hare, and Sponaugle 2007; Tunnicliffe, McArthur, and McHugh 1998; Weersing and Toonen 2009). A variety of biotic factors (such as life‐history strategies and food resources) and abiotic factors (including habitat types, ocean currents, and geographic barriers) have been believed to influence the distribution patterns and adaptive evolution of deep‐sea macrobenthos (Bashevkin et al. 2020; Vrijenhoek 2010).

**FIGURE 1:**
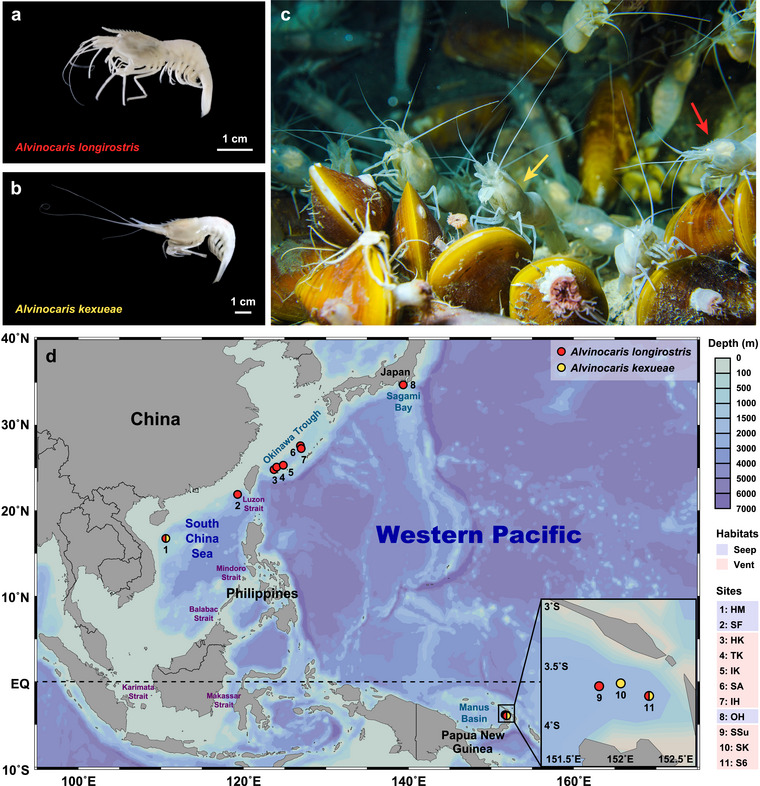
Distribution of *Alvinocaris longirostris* and *A*. *kexueae* in the Western Pacific. (a) A specimen of *A. longirostris* collected from the Site F seep in the South China Sea. (b) A specimen of *A. kexueae* collected from the Haima seep in the South China Sea. (c) A representative photo depicting the co‐occurrence and the natural habitat of *A*. *longirostris* and *A*. *kexueae* in the Haima seep, with *A*. *longirostris* indicated by a red arrow and *A*. *kexueae* by a yellow arrow (photo credit: Dr. Jun Tao from Guangzhou Marine Geological Survey, Guangzhou, China). (d) Populations of *A*. *longirostris* (red dots) and *A*. *kexueae* (yellow dots) were analyzed in this study. Blue and pink shadows below the sites represent seep and vent habitats, respectively. The map was drawn using Ocean Data View (ODV) v.4.6.5 (https://odv.awi.de), with a color bar representing the water depth (m). Abbreviations of populations: 1: HM, Haima seep; 2: SF, Site F seep; 3: HK, Hatoma Knoll vent; 4: TK, Tarama Knoll vent; 5: IK, Irabu Knoll vent; 6: SA, Sakai Field vent; 7: IH, Izena Hole vent; 8: OH, Off Hatsushima seep; 9: SSu, South Su vent; 10: SK, Site “*Kexue*” vent (No site name was provided in the original publication. For easier interpretation, it is tentatively named based on the R/V *Kexue* in this study); 11: S6, Solwara 6 vent.

Deep‐sea shrimps in the family Alvinocarididae (Decapoda: Caridea: Bresilioidea) consist of five extant genera with 36 species, which often dominate chemosynthesis‐based ecosystems, such as hydrothermal vents, hydrocarbon seeps, whale falls, and wood falls (DecaNet eds 2024; Pereira et al. 2020). In the Western Pacific, only three species from the genus *Alvinocaris* have been found in both vent and seep habitats: *A. longirostris* (Figure 1a), *A. kexueae* (Figure 1b), and *A. dissimilis* (He et al. 2023; Yahagi et al. 2015; Zhao et al. 2020). Among these, *A. longirostris* has the widest latitudinal and bathymetric distribution, ranging from 35°N to 3°S and 930 to 1736 m depth (Figure 1d). It inhabits hydrocarbon seeps in the South China Sea and Sagami Bay, as well as hydrothermal vents in the Okinawa Trough and Manus Basin (He et al. 2023; Yahagi et al. 2015; Zhao et al. 2020). In contrast, *A. kexueae* and *A. dissimilis* are found in more limited geographic areas: *A. kexueae* has only been reported from the South China Sea seeps and Manus Basin vents (16°N to 3°S; 1300 to 1910 m depth; Figure 1d; He et al. 2023; Van Audenhaege et al. 2019; Wang and Sha 2017), while *A. dissimilis* has been known exclusively from the Kuroshima Knoll seep in the Ryukyu Arc, the Minami Ensei vent in the Okinawa Trough, and the Higashi Aogashima vent in the Izu‐Ogasawara Arc (32°N to 24°N; 641 to 750 m depth; Methou et al. 2023).

The co‐occurrence of *A*. *longirostris* and *A*. *kexueae* in the South China Sea seeps and the Manus Basin vents (Figure 1c,d), along with their different distribution ranges, raises intriguing questions about how their populations maintain connectivity between widely separated habitats and what factors may have influenced their distinct biogeographical patterns. Studying the population connectivity of these vent‐ and seep‐dwelling species will shed light on their historical colonization, migration dynamics, and genetic divergence. Previous research by Yahagi et al. (2015) detected high genetic homogeneity among *A*. *longirostris* populations in three Okinawa Trough vents and one Sagami Bay seep, based on the mitochondrial *cytochrome c oxidase subunit 1* (*cox1*) gene fragment. However, no population genetic studies have yet been conducted on the recently discovered *A*. *longirostris* populations in the South China Sea seeps and Manus Basin vents (He et al. 2023; Van Audenhaege et al. 2019; Zhao et al. 2020). Furthermore, the lack of population genetic research on *A*. *kexueae* leaves its connectivity and divergence between populations in the South China Sea seep and the Manus Basin vents largely unknown.

To address these issues, we amplified and sequenced the *cox1* gene fragment of *A*. *longirostris* and *A*. *kexueae* from two hydrocarbon seeps in the South China Sea and three vent sites in the Manus Basin. We also included published sequences of more populations in the Western Pacific to conduct a comparative population genetic study (for details, see the Materials and Methods section). Our study aimed to (1) compare the population connectivity of the two *Alvinocaris* species with different distribution ranges, (2) explore the potential drivers that may have shaped their contemporary distribution and genetic divergence, and (3) pave the way for future research to better understand the biogeographic patterns of alvinocaridid shrimps inhabiting chemosynthesis‐based ecosystems worldwide.

## 2 Materials and Methods

### 2.1 Sample Collection, Identification, and Fixation

A total of 64 *Alvinocaris* specimens were collected. Among these, 47 *A. longirostris* individuals were collected from two hydrocarbon seep sites in the South China Sea (Haima seep and Site F) and two vent sites in the Manus Basin (South Su and Solwara 6). Additionally, 17 *A. kexueae* individuals were collected from one seep site in the South China Sea (Haima seep) and two vent sites in the Manus Basin (South Su and Solwara 6) (Figure 1; Table S1). Specimens from the Haima seep were collected using the remotely‐operated vehicle (ROV) *Haima2* onboard the research vessel (R/V) *Haiyang 6* in September 2020 and June 2022. Those from Site F were collected using the ROV *ROPOS* onboard the R/V *Tan Kah Kee* in April and May 2018. Specimens in South Su were collected using the ROV *ST212* onboard the merchant vessel (M/V) *NorSky* in August 2012, and those in Solwara 6 were collected by the same M/V and ROV in July 2008 (Table S1).

After samples were brought to the main deck of the research vessels, species identification was conducted using morphological keys in Kikuchi and Ohta (1995) for *A. longirostris* and Wang and Sha (2017) for *A. kexueae*. The samples were then frozen at −80°C or preserved in 100% ethanol.

### 2.2 DNA Extraction and Molecular Barcoding

The muscle tissue from each individual was dissected for genomic DNA extraction using the DNeasy Blood & Tissue Kit (Qiagen, Germany) according to the manufacturer's protocol. Total genomic DNA was qualified using 1.0% agarose gel electrophoresis and quantified using a NanoDrop ND‐2000 spectrophotometer (Thermo Fisher Scientific, USA). The primer pair LCO1490 and HCO2198 (Folmer, Black, and Vrijenhoek 1994) was used to amplify a fragment of *cox1*. Polymerase chain reactions (PCRs) were performed in a 25‐µL reaction mixture containing 22 µL of PCR mixture from the T3 Super PCR Mix kit (Tsingke Biotech, China), 10 nM primers, and 50 ng genomic DNA. The PCR program included an initial denaturation at 95°C for 180 s, followed by 30 cycles of denaturation at 95°C for 30 s, annealing at 53°C for 30 s, extension at 72°C for 60 s, and a final extension at 72°C for 120 s. The PCR products were sent to Tsingke Biotech (Shenzhen, China) for bi‐directional Sanger sequencing on an ABI 3730 automated DNA sequencer (Applied Biosystems, USA). The obtained *cox1* gene fragments were examined using Chromas v.2.6.5 (Technelysium Pty Ltd; available from http://technelysium.com.au/wp/chromaspro/). The sequences from both directions of the same individual were manually merged into a consensus sequence based on the overlapped bases.

### 2.3 Data Collection and Dataset Processing

In addition to the *cox1* data generated in this study, we included published *cox1* gene fragments from 85 *A. longirostris* individuals and eight *A. kexueae* individuals (Tables S2 and S3) for our population genetic analyses. Among these, sequences from 66 individuals of *A. longirostris* (GenBank accession numbers: LC029871–LC029885) were generated by Yahagi et al. (2015) (Table S2). The remaining 27 sequences were either submitted directly to GenBank or published in various other studies, with their GenBank accession numbers provided in Table S2.

Due to their geographical proximity, we combined *A. longirostris* individuals from the Izena Hole and Sakai Field vents (sites 6 and 7 in Figure 1d, i.e., <30 km) as the Izena Hole and Sakai Field vent population. Similarly, we grouped individuals of *A. longirostris* from the South Su and Solwara 6 vents (sites 9 and 11 in Figure 1d, i.e., <50 km) as the Manus Basin vent population. Additionally, we pooled *A. kexueae* collected from the Site “*Kexue*” and Solwara 6 vents (sites 10 and 11 in Figure 1d, i.e., < 30 km) as the Manus Basin vent population (Table 1).

**TABLE 1:** Genetic diversity of *Alvinocaris longirostris* and *A. kexueae* estimated based on a 622‐bp and 564‐bp fragment of *cox1*, respectively.

Species	Site	*N*	*N*_p_	*N*_h_	*H*	*π* × 10^−2^
*A. longirostris*	All	132	26	31	0.483 ± 0.055	0.108 ± 0.016
	HM	20	6	7	0.521 ± 0.135	0.096 ± 0.030
	SF	19	9	8	0.614 ± 0.130	0.153 ± 0.167
	HK	20	5	5	0.368 ± 0.135	0.080 ± 0.035
	TK	13	4	5	0.539 ± 0.161	0.099 ± 0.035
	IK	20	4	5	0.368 ± 0.135	0.107 ± 0.046
	IS	21	6	7	0.500 ± 0.133	0.092 ± 0.029
	OH	10	3	3	0.378 ± 0.181	0.096 ± 0.053
	MB	9	4	5	0.722 ± 0.159	0.174 ± 0.057
*A. kexueae*	All	25	16	16	0.916 ± 0.041	0.463 ± 0.102
	HM	8	7	7	0.964 ± 0.077	0.362 ± 0.091
	MB	17	12	9	0.823 ± 0.075	0.321± 0.079

Abbreviations: *N*, number of specimens; *N*_p_, number of polymorphic sites; *N*_h_, number of haplotypes; *H*, haplotype diversity (mean ± SD); *π*, nucleotide diversity (mean ± SD); HM, Haima seep; SF, Site F seep; HK, Hatoma Knoll vent; TK, Tarama Knoll vent; IK, Irabu Knoll vent; IS, Izena Hole and Sakai Field vents (sites 6 and 7 in Figure 1d); OH, off Hatsushima seep; MB, Manus Basin vents (sites 9 and 11 for *A. longirostris* in Figure 1d; sites 10 and 11 for *A. kexueae* in Figure 1d).

After pooling the data, we conducted population genetic analyses using *cox1* gene fragments of *A. longirostris* from eight geographical localities. These include two seep populations in the South China Sea (Haima seep; Site F), one seep population in Sagami Bay (Off Hatsushima), four vent populations in the Okinawa Trough (Hatoma Knoll, Tarama Knoll, Irabu Knoll, and Sakai Field and Izena Hole), and one vent population in the Manus Basin (South Su and Solwara 6) (Figure 1d; Table S3).

Additionally, we performed population genetic analyses of *cox1* gene fragments from *A. kexueae*, collected from two geographical localities: one seep population in the South China Sea (Haima seep) and one vent population in the Manus Basin (Site “*Kexue*” and Solwara 6 vents) (Figure 1d; Table S3).

### 2.4 Genetic Statistics

For each species, the *cox1* sequences were aligned using ClustalW (Thompson, Higgins, and Gibson 1994) implemented in MEGA v.11 (Tamura, Stecher, and Kumar 2021) under the default settings, and then manually trimmed to the same length. The genetic dissimilarity was assessed by calculating the Kimura 2‐parameter (K2P) distance (Kimura 1980) implemented in MEGA v.11 (Tamura, Stecher, and Kumar 2021), based on a pairwise comparison of the shared nucleotide bases. These results were then illustrated using the “HeatMap” function implemented in TBtools v.2.030 (Chen et al. 2023).

We also estimated four genetic statistics, including polymorphic sites (*N*_p_), haplotypes (*N*_h_), haplotype diversity (*H*), and nucleotide diversity (*π*) (Nei 1987), for each population of each species using DnaSP v.6.12.03 (Rozas et al. 2017).

### 2.5 Genetic Differentiation and Population Divergence

To quantify the genetic differentiation between populations of each species, the pairwise fixation index (*F*_ST_) (Hudson, Slatkin, and Maddison 1992) was estimated using Arlequin v.3.5.2 (Excoffier and Lischer 2010), running 1000 permutations to test for statistical significance. To unveil the potential genetic structure of each species, a TCS network was constructed using PopART v.1.7 (Clement, Posada, and Crandall 2000; Leigh, Bryant, and Nakagawa 2015), and a Bayesian clustering approach was applied using STRUCTURE v.2.3.4 (Pritchard, Stephens, and Donnelly 2000). For the STRUCTURE analysis, the number of genetic groups (*K*) was predefined from 1 to 11 for *A. longirostris* and 1 to 5 for *A. kexueae* based on the number of their respective sampling localities. Seven runs were executed for each *K* value, with each run applying a burn‐in of 10 000 followed by 100 000 Markov chain Monte Carlo (MCMC) iterations. The most probable *K* was evaluated using the Δ*K* method implemented in STRUCTURE HARVESTER (Earl and VonHoldt 2012; Evanno, Regnaut, and Goudet 2005). An optimal alignment of all replicate runs of the most probable *K* was obtained using CLUMPP v.1.1 (Jakobsson and Rosenberg 2007) and then visualized using DISTRICT v.1.1 (Rosenberg 2004). All analyses were performed for each species based on the *cox1* sequences of all individuals.

### 2.6 Migration Rate

Migration rates between populations of each species were estimated using MIGRATE‐N v.3.7.2 based on a Bayesian method (Beerli and Palczewski 2010). Uniform priors were specified for both the effective population size (Θ) (minimum = 0; maximum = 0.1) and the mutation‐scaled immigration rate (M) (minimum = 0, maximum = 1000; delta = 100). We performed three independent runs and set static heating with four heating chains (1, 1.5, 3, 100 000). For each independent run, a sampling increment of 20 was applied with a total of 50 000 recorded steps in the chain after discarding an initial burn‐in of 10 000.

### 2.7 Demographic History

To explore the demographic history of each species, we estimated three neutrality statistics, including Tajima's *D* (Tajima 1989), Fu's *F*_s_ (Fu 1997), and Ramos‐Onsins and Rozas's *R*_2_ (*R*_2_) (Ramos‐Onsins and Rozas 2002), using DnaSP v.6.12.03 (Rozas et al. 2017) for all individuals and the identified genetic groups. Among them, Ramos‐Onsins and Rozas's *R*_2_ is considered the most powerful for populations with a small sample size (Ramos‐Onsins and Rozas 2002). Significant negative values for both Tajima's *D* (Tajima 1989) and Fu's *F*_s_ (Fu 1997), as well as a significant small positive value for Ramos‐Onsins and Rozas's *R*_2_ (*R*_2_) (Ramos‐Onsins and Rozas 2002), all indicate recent population expansion, but similar results can also be a consequence of selective sweep, especially for Tajima's *D* and Fu's *F*_s_ (Fu 1997; Tajima 1989).

Furthermore, a mismatch distribution analysis implemented in Arlequin v.3.5.2 (Excoffier and Lischer 2010) was performed to assess the expansion of each species for all individuals and the identified genetic groups by linear fitting the observed and expected curves calculated using the Harpending's raggedness index (*Hri*; Harpending 1994), running 10 000 bootstraps to test for statistical significance. A non‐significant and positive *Hri* value supports the null hypothesis, indicating a recent expansion in population size (Harpending 1994; Kanginakudru et al. 2008).

The demographic history of each species was also inferred using a Bayesian skyline plot (BSP) analysis implemented in BEAST v.2.5.0 (Bouckaert et al. 2014) for all individuals and the identified genetic groups. Due to the lack of data on the mutation rate of *cox1* and the generation time for *A. longirostris* and *A. kexueae*, we assumed an estimated mutation rate of 1.4% per million years (Gonzalez‐Castellano et al. 2020; Knowlton and Weigt 1998) and a 2‐year generation time for both species (Copley and Young 2006). The optimal clock model, as outlined in Table S4 and identified by the highest Bayes factor according to Baele et al. (2012), was chosen for constructing the BSPs. We ran the analysis for one billion MCMC iterations, sampling every 1000 iterations and discarding the first 10% as burn‐in. To assess the BSPs, we used Tracer v.1.7.1 (Rambaut et al. 2018) and ensured that the effective sample size (ESS) values were greater than 200.

## 3 Results

### 3.1 Population Diversity

**FIGURE 2:**
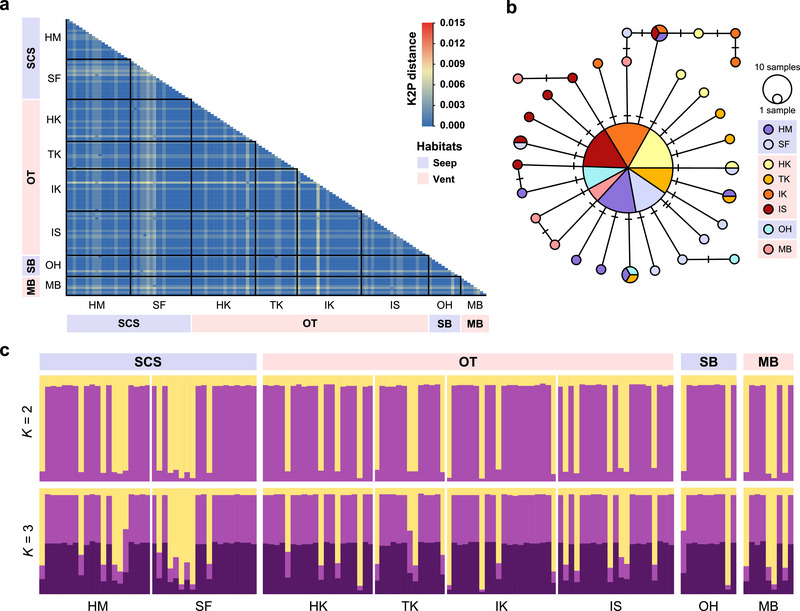
Genetic divergence of *Alvinocaris longirostris* based on a 622‐bp fragment of *cox1*. (a) The heatmap of pairwise Kimura 2‐parameter (K2P) distance. Individuals from the same population are outlined by black squares. (b) The TCS network of *cox1* haplotypes. The number of hatch marks along the edges indicates nucleotide substitutions. Each circle represents one haplotype, with different colors representing the population where the haplotypes were found, while black circles indicate unknown or missing haplotypes. The size of the circle is proportional to the frequency of the haplotype. (c) Population structure at *K* = 2 and *K* = 3. Each individual is represented by a vertical bar, with different colors indicating the ancestry of different genetic groups. Blue and pink shadows below the sites and regions indicate the seep and vent habitats, respectively. Abbreviations: SCS, South China Sea; OT, Okinawa Trough; SB, Sagami Bay; MB, Manus Basin (vents: sites 9 and 11 in Figure 1d); HM, Haima seep; SF, Site F seep; HK, Hatoma Knoll vent; TK, Tarama Knoll vent; IK, Irabu Knoll vent; IS, Izena Hole and Sakai Field vents; OH, Off Hatsushima seep.

For *A. longirostris*, the *cox1* gene fragments of 47 individuals were sequenced in this study and deposited in GenBank with the following accession numbers: OR048141–OR048155, PP838258–PP838261, PP838272–PP838299, and PP838455–PP838457 (Table S3). When combined with the published data, alignment and trimming resulted in 622‐bp *cox1* fragments from 132 *A. longirostris* individuals. These fragments exhibited low K2P genetic distances, ranging from 0 to 0.00646, for individuals from different localities (Figure 2a; Table S5). We identified a total of 31 *cox1* haplotypes, yielding a mean haplotype diversity (*H*) of 0.482 ± 0.055 and a mean nucleotide diversity (*π*) of 0.00108 ± 0.00016 (Table 1). The *H* and *π* values for each population ranged from 0.368 to 0.722 and 0.00092 to 0.00174, respectively (Table 1). Among all examined localities, those inhabiting the Site F seep in the South China Sea and the Manus Basin vent exhibited slightly higher *H* (0.614 ± 0.130 and 0.722 ± 0.159, respectively) and *π* (0.153 ± 0.167 and 0.174 ± 0.057, respectively) compared to other locations.

**FIGURE 3:**
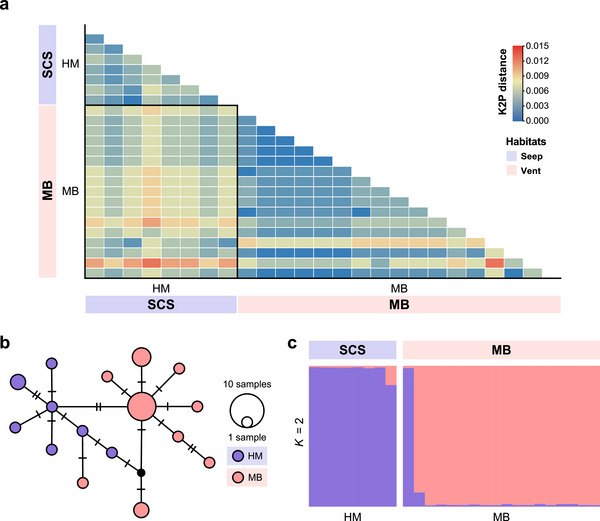
Genetic divergence of *Alvinocaris kexueae* estimated based on a 564‐bp fragment of *cox1*. (a) The heatmap of pairwise Kimura 2‐parameter (K2P) distance. Individuals from the same population are outlined by black squares. (b) The TCS network of *cox1* haplotypes. The number of hatch marks along edges indicates the number of nucleotide substitutions. Each circle represents one haplotype, with each color representing the population where the haplotype was found, while black circles indicate unknown or missing haplotypes. The size of the circle is proportional to the frequency of the haplotype. (c) Population structure at *K* = 2. Each individual is represented by a vertical bar with different colors indicating the ancestry of different genetic groups. Blue and pink shadows below the sites and regions were used to illustrate seep and vent habitats, respectively. Abbreviations: SCS, South China Sea; HM, Haima seep; MB, Manus Basin (vents: sites 10 and 11 for in Figure 1d).

For *A. kexueae*, we generated the *cox1* gene fragments from 17 individuals, which have been deposited in GenBank with the following accession numbers: OR048133–OR048139, PP838216, PP838217, PP838458, and PP838459 (Table S3). After aligning with published data and trimming the sequences, we retained 564‐bp *cox1* gene fragments from 25 *A. kexueae* individuals for further analysis. The *cox1* sequences from the two examined populations showed low K2P genetic distances, ranging from 0 to 0.01253 (Figure 3a; Table S7). We identified a total of 16 *cox1* haplotypes, resulting in a mean *H* of 0.916 ± 0.041 and a mean *π* of 0.00463 ± 0.00102 (Table 1). Notably, *A*. *kexueae* collected from the Haima seep in the South China Sea exhibited higher *H* (0.964 ± 0.077 compared to 0.823 ± 0.075) and *π* (0.00362 ± 0.00091 compared to 0.00321 ± 0.00079) than those from the Manus Basin vents.

### 3.2 Genetic Differentiation Between Populations

For *A*. *longirostris*, pairwise *F*_ST_ estimation revealed no obvious genetic differentiation between populations in the Western Pacific, with values ranging from −0.00695 to −0.07769). However, statistical significance was detected between the Haima seep population in the South China Sea and the Manus Basin vent population (*F*_ST_ = 0.05176, *p* < 0.05), as well as between the Hatoma Knoll vent population in the Okinawa Trough and the Manus Basin vent population (*F*_ST_ = 0.06189, *p* < 0.05; Table 2). The TCS network displayed a star‐shaped structure, with the most prevalent *cox1* haplotype shared among the eight populations at the center and surrounded by several low‐frequency and private haplotypes (Figure 2b). Similarly, the STRUCTURE analysis indicated that *A*. *longirostris* functions as one metapopulation, exhibiting a shared ancestry pattern with no clear genetic subdivision among individuals from all eight localities, although the most probable value of *K* was 3 (Figure 2c).

**TABLE 2:** Pairwise *F*_ST_ statistics estimated between populations of *Alvinocaris longirostris* (below diagonal) and *A. kexueae* (above diagonal) based on a 622‐bp and a 564‐bp fragment of *cox1*, respectively.

Site	MB	HM	SF		HK	TK	IK	IS
**MB**	—	0.46350^***^	—	—		—	—	—
**HM**	0.05176^*^	—	—	—		—	—	—
**SF**	−0.00138	0.00399	—	—		—	—	—
**HK**	0.06189^*^	0.00000	−0.00653	—		—	—	—
**TK**	0.03799	−0.02568	−0.00695	0.00294		—	—	—
**IK**	0.07769	0.01914	0.01517	0.00107		0.02283	—	—
**IS**	0.05059	−0.00417	−0.00217	0.00006		0.00396	0.02157	—
**OH**	0.02908	−0.01695	−0.03943	−0.00455		−0.02625	0.02570	0.00648

Abbreviations: MB, Manus Basin vents (sites 9 and 11 for *A. longirostris* in Figure 1d; sites 10 and 11 for *A. kexueae* in Figure 1d); HM, Haima seep; SF, Site F seep; HK, Hatoma Knoll vent; TK, Tarama Knoll vent; IK, Irabu Knoll vent; IS, Izena Hole and Sakai Field vents (sites 6 and 7 in Figure 1d); OH, off Hatsushima seep.

Significance: ^*^ 0.01 < *p* ≤ 0.05; ^**^ 0.001 < *p* ≤ 0.01; ^***^ *p* ≤ 0.001.

For *A*. *kexueae*, pairwise *F*_ST_ estimation revealed significant genetic differentiation between the Haima seep and Manus Basin vent populations (*F*_ST_ = 0.46350, *p* < 0.001; Table 2). Notably, no *cox1* haplotype was shared between individuals from these two populations. The *cox1* haplotypes from the Manus Basin vents differed from those of the Haima seep by one to seven nucleotide substitutions (Figure 3b). The STRUCTURE analysis supported this finding, indicating two distinct genetic groups (the most probable *K* value = 2) that corresponded to their sampling localities (Figure 3c).

### 3.3 Gene Flow Between Populations

For *A*. *longirostris*, the migration analysis revealed extensive gene flow between most population pairs, with moderate to high migration rates from 3.384 to 25.941 (Table S6). A distinct pattern of northward gene flow was observed, primarily from other populations toward the Off Hatsushima seep in Sagami Bay, with rates between 3.384 and 21.200. In contrast, migration in the opposite direction was much lower, ranging from 0.232 to 0.258 (Table S6).

For *A*. *kexueae*, the migration analysis showed stronger gene flow from the Haima seep to the Manus Basin vents compared to the reverse direction (7.829 vs. 3.970; Table S8). This finding aligned with those obtained from the STRUCTURE analysis, where one individual from the Manus Basin vents exhibited significant genetic ancestry from the Haima seep (Figure 3c).

### 3.4 Demographic History

**FIGURE 4:**
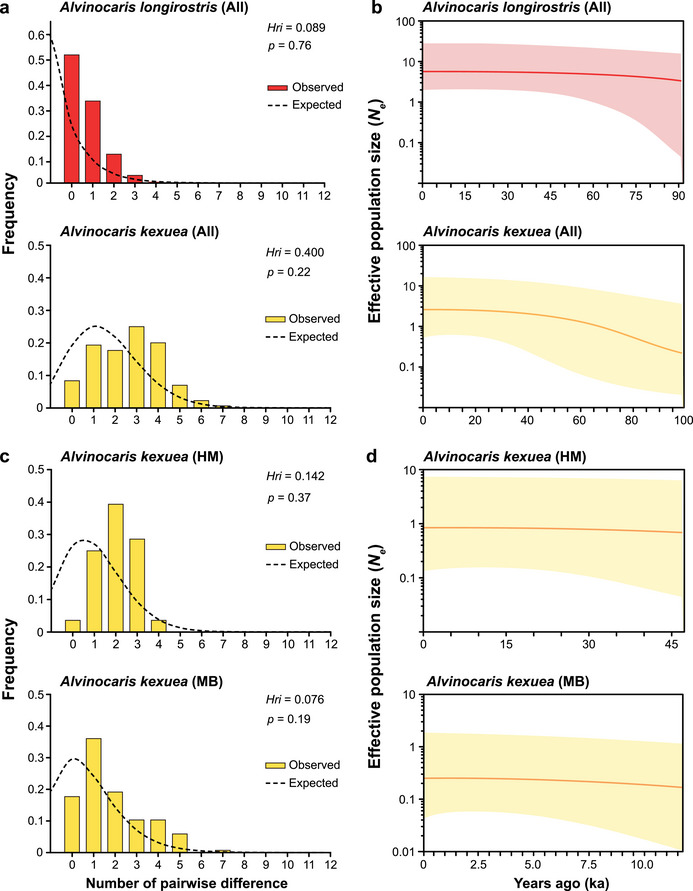
Demography of *Alvinocaris longirostris* and *A. kexueae* inferred based on a 622‐bp and a 564‐bp fragment of *cox1*, respectively. (a) Mismatch distributions for all individuals of both species. (b) Bayesian skyline plots (BSPs) for all individuals of both species. (c) Mismatch distributions and (d) BSPs for the Haima (HM) seep and the Manus Basin (MB) vent genetic groups of *A. kexueae*. In (a) and (c), the observed mismatch distributions are denoted by vertical bars, and the expected distribution under the population expansion model is represented by a dashed line. In (b) and (d), the horizontal axis represents backward time in kilo years (ka), and the vertical axis indicates the generation‐scaled effective population size (*N*_e_). The median estimate of the generation‐scaled *N*_e_ is depicted by a solid line, with a shadow showing its upper and lower bounds of the 95% highest probability density (HPD) interval. Abbreviation: *Hri*, Harpending's raggedness index.

For *A*. *longirostris*, both Tajima's *D* and Fu's *F*_s_ values for the metapopulation, which included all individuals, were negative with statistical significance. In contrast, the values of Ramos‐Onsins and Rozas’ *R*_2_ were small and positive with statistical significance (Table 3). The mismatch distribution analysis for the metapopulation showed a skewed distribution (Figure 4a). Additionally, the median generation‐scaled effective population size (*N*_e_) during the recent demographic history of *A*. *longirostris*, as revealed by BSPs, was relatively stable. However, the 95% highest probability density (HPD) interval showed an increasing trend in *N*_e_ (Figure 4b).

**TABLE 3:** Demographic statistics for *Alvinocaris longirostris* and *A*. *kexueae* calculated based on a 622‐bp and a 564‐bp fragment of *cox1*, respectively.

Species	Genetic group	Neutral test		
		*D*	*F*_s_	*R*_2_
*A. longirostris*	All	−2.566^**^	−53.075^***^	0.0161^*^
*A. kexueae*	All	−1.352^*^	−11.123^***^	0.0686^*^
	MB	−1.840^*^	−4.374^**^	0.0773^*^
	HM	−1.170	−4.477^**^	0.1019^**^

Abbreviations: All, all individuals; MB, Manus Basin vent genetic group; HM, Haima seep genetic group; *D*, Tajima's *D*; *F*_s_, Fu's *F*_s_; *R*_2_, Ramos–Onsins and Rozas's *R*_2_.

Significance: ^*^ 0.01 < *p* ≤ 0.05; ^**^ 0.001 < *p* ≤ 0.01; ^***^ *p* ≤ 0.001.

For *A. kexueae*, both Tajima's *D* and Fu's *F*_s_ values for all individuals pooled from all genetic populations and its Manus Basin vent genetic group were negative with statistical significance. In contrast, the Ramos‐Onsins and Rozas's *R*_2_ values were both small but positive with statistical significance (Table 3). Due to the small sample size of only eight individuals, the Tajima's *D* value for the Haima seep genetic group was negative but not statistically significant (Table 3). However, Fu's *F*_s_ value for this group was negative and the Ramos‐Onsins and Rozas's *R*_2_ value was small and positive, both showing statistical significance (Table 3). The mismatch distribution analyses for all individuals (Figure 4a) and the two genetic groups (Figure 4c) consistently produced a unimodal distribution. Moreover, all individuals of *A. kexueae* (Figure 4b) and its two genetic groups (Figure 4d) exhibited an increasing trend in *N*_e_ during their recent evolutionary history; however, such increase was more noticeable in the BSP of all individuals compared to that of the two genetic groups.

## 4 Discussion

In this study, we investigated the genetic diversity and population connectivity of two *Alvinocaris* species, *A. longirostris* and *A. kexueae*, which inhabit both vent and seep habitats in the Western Pacific. We found that the more widely distributed species, *A*. *longirostris* (ranging from 35°N to 3°S and at depths of 930 to 1736 m), exhibited lower genetic differentiation than the more narrowly distributed species, *A. kexueae* (found between 16°N and 3°S at depths of 1300 to 1910 m depth). Our analyses, including *F*_ST_ estimation, TCS network reconstruction, and STRUCTURE analysis, indicated that the eight disjoint populations of *A*. *longirostris* (comprising two seep populations in the South China Sea, one seep population in the Sagami Bay, four vent populations in the Okinawa Trough, and one vent population in the Manus Basin) were genetically well‐mixed, functioning as one metapopulation. In contrast, *A. kexueae* showed clear genetic differentiation between its Haima seep population in the South China Sea and the Manus Basin vent population. Despite their differences in genetic diversity and population divergences, results of three neutral tests, mismatch distribution analyses, and BSP construction consistently implied that both *A*. *longirostris* and *A. kexueae* may have experienced population expansion during their recent evolutionary history.

### 4.1 Potential Factors Shaping Distribution and Biogeography

One of the key factors influencing the distribution ranges and biogeographic patterns of deep‐sea macrobenthos is the duration of their pelagic larvae and their larval dispersal behavior (Arellano et al. 2014; Tunnicliffe, McArthur, and McHugh 1998). Previous morphological observations (Hernández‐Ávila, Cambon‐Bonavita, and Pradillon 2015; Ramirez‐Llodra and Segonzac 2006), along with analyses of fatty acid biomarkers and stable isotope analyses (Stevens et al. 2008), have shown that alvinocaridid shrimps produce larvae with well‐developed eyes. These larvae exhibit primary lecithotrophy in their early large stages, obtaining nutrients from the egg yolk, followed by a planktotrophic phase in which they feed on plankton during later larval development. Physiological experiments indicate that *A. longirostris* can hatch within a temperature range of 5°C to 20°C. The hatched larvae swim upward to surface layers and can survive for up to 88 days at 10°C under atmospheric pressure (Watanabe et al. 2016). These findings suggest that *A. longirostris* has an extended pelagic larval duration and the capability to migrate vertically into upper ocean currents for long‐distance dispersal. Our TCS network reconstruction and STRUCTURE analysis further support this idea, indicating that *A. longirostris* forms a single panmictic metapopulation across eight disjoint geological localities. Additionally, our estimation of migration rates revealed directional migration of *A*. *longirostris* from other populations toward Sagami Bay, consistent with the trajectory of the Kuroshio Current. The Kuroshio Current is a strong western boundary current that flows from the equatorial region to the Northwest Pacific, extending from the surface to approximately 200 m deep, with influence that can reach depths exceeding 1000 m (Su, Guan, and Jiang 1990). In summary, it is likely that the larvae of *A. longirostris* primarily disperse through the upper water layer, including the Kuroshio Current, to achieve long‐distance migration and extensive genetic exchange among individuals from island‐like vent and seep habitats.

Although physiological investigations of *A. kexueae* have not yet been conducted, our results suggest that this species may have a shorter pelagic larval duration and/or different dispersal behaviors compared to *A. longirostris*. For example, *A. kexueae* is found in the Haima seep and the Manus Basin vents, both of which are located in regions with relatively high sea surface temperatures. This indicates that its larval dispersal may be constrained by a narrower water temperature range (Zhang, Pagani, and Liu 2014). Previous studies have shown that several alvinocaridid species, such as *A. longirostris*, *Shinkaicaris leurokolos*, and *Rimicaris loihi*, exhibit distinct thermal preferences for brooding and larval dispersal (Methou et al. 2023; Watanabe et al. 2016). It is possible that *A. kexueae* and *A. longirostris* also exhibit different thermal preferences. To confirm this hypothesis, further validation through *in situ* observation and *ex situ* rearing experiments of *A. kexueae* under controlled water temperature and pressure would be necessary.

Another potential explanation for the differing distribution patterns and genetic divergence between *A. longirostris* and *A. kexueae* is their adaptation to local environments. Both deep‐sea vent and seep habitats are chemosynthesis‐based ecosystems characterized by darkness and high pressure, nevertheless. However, their fluids differ in several properties, including flow rates, temperatures, and geochemical compositions (Levin et al. 2016). While our population genetic analyses relied on only one mitochondrial gene, our results indicate that the vent and seep populations of *A. longirostris* exhibit high genetic homogeneity, whereas *A. kexueae* populations show significant genetic divergence. This suggests that local environmental factors may have played a more pronounced role in shaping the genetic differentiation of *A. kexueae* compared to *A. longirostris*. However, it remains unclear whether the identified divergence in *A. kexueae* is primarily driven by selective (e.g., local adaptation) or neutral processes (e.g., limited connectivity). Additional in‐depth investigations, especially through the larval rearing experiments and the implementation of genome‐wide and/or transcriptome‐wide single nucleotide polymorphisms (SNPs) generated from high throughput sequencing approaches are necessary to better address this issue (Allendorf 2017).

Additionally, feeding preference may have also contributed to the distinct distribution patterns of *A. longirostris* and *A. kexueae* (Yahagi et al. 2015). Like *Rimicaris*, which exhibits ontogenetic and interspecies differences in dietary preferences (Methou et al. 2020), the larvae and adults of *A. kexueae* might possess a specialized diet compared to *A. longirostris*, and this could influence their dispersal depth and restrict their distribution to areas where the preferred food sources are abundant (Stevens et al. 2008; Yahagi et al. 2015, 2017). However, this interpretation requires careful examination, as *Alvinocaris* species are generally considered opportunistic feeders capable of consuming a variety of microbes and macrobenthos (Gebruk et al. 2000; Van Audenhaege et al. 2019; Zelnio and Hourdez 2009).

### 4.2 Facilitation of Long‐Distance Dispersal by Ocean Currents and Intermediate Habitats

The potential dispersal of *A. longirostris* larvae in the upper water layers may be influenced by intrusions of the Kuroshio Current and the North Pacific Intermediate Water into the South China Sea through the Luzon Strait. These currents could contribute to the high genetic connectivity of *A. longirostris* populations on both sides of the strait (Nan, Xue, and Yu 2015; You et al. 2005). A similar situation has been observed in the deep‐sea mussel *Gigantidas platifrons*, which inhabits areas similar to those of *A. longirostris* and is believed to release planktotrophic larvae that likely disperse in the upper ocean currents (Xu et al. 2018).

The potential dispersal pathway for *A. kexueae* (Haima seep) and *A*. *longirostris* (Haima seep and Site F) from the South China Sea seeps to the Manus Basin vents and vice versa remains uncertain. However, the South China Sea Throughflow, which involves the inflow through the Luzon Strait and the outflow through the Mindoro, Balabac, Karimata, and Makassar Straits, may play a role in facilitating this process (Gordon et al. 2012; Qu, Song, and Yamagata 2009). Given the significant geographic distance between the South China Sea and Manus Basin, the presence of ancient seep deposits in the Philippines (Kiel, Aguilar, and Kase 2020; Majima et al. 2007) and active vents in the Sunda Arc (McConachy, Binns, and Permana 2001; Wiedicke et al. 2002), along with the co‐occurrence of other deep‐sea taxa alongside the two studied alvinocaridids in both areas (e.g., Cheng et al. 2024; Thomas et al. 2020), it is likely that unreported intermediate stepping‐stone sites exist between the South China Sea seeps and Manus Basin vents. These sites could facilitate larval dispersal and genetic exchange between *A. kexueae* and *A*. *longirostris* across the distant regions separating the two areas, similar to what has been inferred for another alvinocarid species, *Alvinocaris muricola*, in the Atlantic Ocean (Pereira et al. 2020).

Future interdisciplinary studies involving systematic deep‐sea surveys, population genomic analyses, and biophysical modeling will provide deeper insights into the migration dynamics of these species in the Western Pacific.

### 4.3 Recent Population Expansion

Although *A. longirostris* and *A. kexueae* exhibit different distribution ranges, levels of genetic divergence, and patterns of migration, our results from neutral tests, mismatch distributions, and BSPs suggest that both species may have experienced recent bottlenecks followed by expansions. This phenomenon has been documented in alvinocaridid shrimps (Pereira et al. 2020; Teixeira et al. 2011, 2013) and other taxonomic groups, including the limpet *Lepetodrilus elevatus*, which inhabits vent fields along the southern East Pacific Rise (Plouviez et al. 2009), the scaly‐foot gastropod *Chrysomallon squamiferum* found in Indian Ocean vent fields (Chen et al. 2015), and the provannid snail *Ifremeria nautilei* living in Southwest Pacific vent fields (Tran Lu et al. 2022). Previous studies have suggested that such population expansions in deep‐sea animals are likely linked to their secondary colonization of new sites or the ephemeral nature of their habitats (Plouviez et al. 2009; Vrijenhoek 2010).

However, due to the use of one single marker gene with an unknown mutation rate and the lack of information on the generation times of *A. longirostris* and *A. kexueae*, we were unable to determine the timing and causes of their potential demographic shifts. Further population genomic studies that incorporate more data will provide valuable insights into this issue.

## 5 Conclusion and Perspectives

Overall, this study introduces new localities and significantly expands the *cox1* database of *A. longirostris* beyond the samples analyzed by Yahagi et al. (2015), which focused on three vents in the Okinawa Trough vents and one seep in Sagami Bay. Additionally, our research enhances the understanding of the genetic diversity and population connectivity of *A. longirostris*, a wider‐distributed alvinocaridid species, and *A. kexueae*, a species with a more restricted distribution, although both species have an overlapping distribution range. Our findings regarding two congeneric species with differing patterns of genetic divergence have important conservation implications, particularly for *A. kexueae*. This species has isolated populations in the South China Sea and Manus Basin that are not connected by contemporary gene flow. Disturbances such as mining for rare metals from vents (Okamoto et al. 2019) and the extraction of gas hydrate from seeps (Zhang et al. 2021) could lead to local extinction, since recent long‐distance larval dispersal for *A. kexueae* is unlikely. Therefore, developing a protection plan for these shrimp populations against anthropogenic disturbances will require a detailed analysis of the population genetics of each species. Our findings will provide a genetic foundation for future research that employs multiple gene markers (e.g., Breusing, Johnson, and Tunnicliffe 2015; Xi et al. 2023) or even SNPs obtained from high throughput sequencing techniques (e.g., Xu et al. 2021, 2024). These efforts will provide deeper insights into the demographic history, biogeographic mechanisms, and genetic adaptations of alvinocaridid shrimps thriving in chemosynthesis‐based ecosystems worldwide.

## Conflicts of Interest

The authors declare no conflicts of interest.

## Supporting information




**Table S1** Sampling information of *Alvinocaris longirostris* and *A. kexueae* in the Western Pacific
**Table S2** Mitochondrial *cox1* genes of *Alvinocaris longirostris* and *A. kexueae* available in public databases included for population genetic analyses in this study
**Table S3** GenBank accession numbers for the mitochondrial *cox1* genes of *Alvinocaris longirostris* and *A. kexueae* generated in this study
**Table S4** Marginal likelihoods estimated based on different clock and prior tree models for the Bayesian skyline plot (BSP) analysis
**Table S5** Pairwise Kimura 2‐parameter (K2P) distance of *Alvinocaris longirostris*
**Table S6** Migration rates between *Alvinocaris longirostris* populations estimated based on a fragment of 622‐bp mitochondrial *cox1* gene using MIGRATE
**Table S7** Pairwise Kimura 2‐parameter (K2P) distances of *Alvinocaris kexueae*
**Table S8** Migration rates between *Alvinocaris kexueae* populations estimated based on a fragment of 564‐bp mitochondrial *cox1* gene using MIGRATE
