# Supplementary material for: Comparative Population Genetics of Two Alvinocaridid Shrimp Species in Chemosynthetic Ecosystems of the Western Pacific
Source: Integr Zool. 2025 Jan 27;21(2):291–303. doi: 10.1111/1749-4877.12954 (PMC12971625; doi:10.1111/1749-4877.12954)
Supplement: Supplementary file 1 — Table S1 Sampling information of Alvinocaris longirostris and A. kexueae in the Western Pacific Table S2 Mitochondrial cox1 genes of Alvinocaris longirostris and A. kexueae available in public databases included for population genetic analyses in this study Table S3 GenBank accession numbers for the mitochondrial cox1 genes of Alvinocaris longirostris and A. kexueae generated in this study Table S4 Marginal likelihoods estimated based on different clock and prior tree models for the Bayesian skyline plot (BSP) analysis Table S5 Pairwise Kimura 2‐parameter (K2P) distance of Alvinocaris longirostris Table S6 Migration rates between Alvinocaris longirostris populations estimated based on a fragment of 622‐bp mitochondrial cox1 gene using MIGRATE Table S7 Pairwise Kimura 2‐parameter (K2P) distances of Alvinocaris kexueae Table S8 Migration rates between Alvinocaris kexueae populations estimated based on a fragment of 564‐bp mitochondrial cox1 gene using MIGRATE [file INZ2-21-291-s001.pdf]

Supplementary Tables

This file contains eight tables with each table being presented in one data sheet.

No. Table	Captions
S1	Sampling information of *Alvinocaris longirostris* and *A. kexueae* in the Western Pacific.
S2	Mitochondrial *cox1* genes of *Alvinocaris longirostris* and *A . kexueae* available in public databases included for population genetic analyses in this study.
S3	Genebank accession numbers for the mitochondrial *cox1* genes of *Alvinocaris longirostris* and *A. kexueae* generated in this study.
S4	Marginal likelihoods estimated based on different clock and prior tree models for the Bayesian skyline plot (BSP) analysis.
S5	Pairwise Kimura 2-parameter (K2P) distance of *Alvinocarid longirostris* in this study.
S6	Migration rates between *Alvinocaris longirostris* populations estimated based on a fragment of 622-bp mitochondrial *cox1* gene using MIGRATE.
S7	Pairwise Kimura 2-parameter (K2P) distance of *Alvinocaris kexueae* in this study.
S8	Migration rates between *Alvinocaris kexueae* populations estimated based on a fragment of 564-bp mitochondrial *cox1* gene using MIGRATE.

Table S1 Sampling information of *Alvinocaris longirostris* and *A. kexueae* in the Western Pacific.

Region	Site	Habitat	Coordinate	Depth (m)	Species	Sample size	Vessel	Submersible	Date
South China Sea	Haima seep	Seep	16°43.8’N, 110°28.5’E	1,300–1,400	*A. longirostris*	15	R/V *Haiyang 6*	ROV *Haima2*	September, 2020
					A. kexueae	7			
					A. longirostris	5	R/V *Haiyang 6*	ROV *Haima2*	June, 2022
					A. kexueae	1			
	Site F	Seep	22°07.0’N, 119°17.0’E	1,120–1,155	*A. longirostris*	18	R/V *Tan Kah Kee*	ROV *ROPOS*	April to May, 2018
Manus Basin	Solwara 6	Vent	3°43.667’S, 151°40.869’E	1,710	*A. longirostris*	1	M/V *NorSky*	ROV *ST212*	July, 2008
					A. kexueae	9			
	South Su	Vent	3°48.404’S, 152°06.298’E	1,295	*A. longirostris*	8			August, 2012

Notes: ROV, remote-operated vehicle; R/V, research vessel; M/V, merchant vessel.

Table S2 Mitochondrial *cox1* genes of *Alvinocaris longirostris* and *A. kexueae* available in public databases included for population genetic analyses in this study.

No.	Species	GenBank Accession No.	Length of seq (bp)	Region	Site	Habitat	Coordinate	Depth (m)	Reference
1	*A. longirostris*	MN807251	1,535	OT	SA	Vent	27°31.475'N, 126°59.021' E	1,550	[1]
2	*A. longirostris*	NC_042497	1,534	OT	HK	Vent	27°32.700'N, 126°58.200'E	1,360	Tokuda et al. (2019) directly submitted to NCBI
3	*A. longirostris*	AB821296	1,534	OT	HK	Vent	27°32.700'N, 126°58.200'E	1,360	[2]
4	*A. longirostris*	AB222051	672	SB	OH	Seep	35°00.000' N, 139°13.000'E	930	[3]
5	*A. longirostris*	AB222050	672						
6	*A. longirostris*	LC029880	658						[4]
7	*A. longirostris*	LC029871	658	OT	IK	Vent	25°14.378'N, 124°52.863'E	1,650	
8	*A. longirostris*	LC029872	658						
9	*A. longirostris*	LC029873	658						
10	*A. longirostris*	LC029874	658						
11	*A. longirostris*	LC029875	658						
12	*A. longirostris*	LC029876	658	OT	HK	Vent	24°51.000'N, 123°50.000'E	1,470	
13	*A. longirostris*	LC029877	658						
14	*A. longirostris*	LC029878	658						
15	*A. longirostris*	LC029879	658						
16	*A. longirostris*	LC029881	658	OT	IH	Vent	27°16.296'N, 127°04.884'E	1,600	
17	*A. longirostris*	LC029882	658						
18	*A. longirostris*	LC029883	658						
19	*A. longirostris*	LC029884	658						
20	*A. longirostris*	LC029885	658						
21	*A. longirostris*	OP482475	724	OT	TK	Vent	25°5.486'N, 124°32.808'E	1,736	Methou et al. (2022) directly submitted to NCBI
22	*A. longirostris*	OP482476	724						
23	*A. longirostris*	OP482477	724						
24	*A. longirostris*	OP482478	724						
25	*A. longirostris*	OP482479	731						
26	*A. longirostris*	OP482480	731						
27	*A. longirostris*	OP482481	731						
28	*A. longirostris*	OP482482	724						
29	*A. longirostris*	OP482483	724						
30	*A. longirostris*	OP482484	731						
31	*A. longirostris*	OP482485	731						
32	*A. longirostris*	OP482486	724						
33	*A. longirostris*	OP482487	731						
34	*A. longirostris*	MT772314	632	SCS	SF	Seep	22°6.930'N, 119°17.112'E	1,120–1,155	[5]
35	*A. kexueae*	MH714459	1,534	MB	SK	Vent	3°42.261'S, 151°52.665'E	Unavailable	Sun & Sha (2018) directly submitted to NCBI
36	*A. kexueae*	MH398098	662	MB	SK	Vent	3°42.261'S, 151°52.665'E	Unavailable	[6]
37	*A. kexueae*	KX825833	658	MB	SK	Vent	3°42.261'S, 151°52.665'E	1,910	[7]
38	*A. kexueae*	KX825834	658						
39	*A. kexueae*	KX825835	658						
40	*A. kexueae*	MK671473	607	MB	SSu	Vent	3°48.404'S, 152°6.298'E	1,324	[8]
41	*A. kexueae*	MK671475	538						
42	*A. kexueae*	MK671476	597						

Notes: OT, Okinawa trough; SB, Sagami Bay; SCS, South China Sea; MB, Manus Basin; SF, Site F seep; HK, Hatoma Knoll vent; TK, Tarama Knoll vent; IK, Irabu Knoll vent; IS, Izena Hole vent; SA, Sakai Field vent; OH, Off Hatsushima seep; SSu, South Su vent; SK, Site Kexue (Site 10 in Fig.1d; No site name was provided in the original publication. For easier interpretation, it is tentatively named based on the R/V *Kexue*).

References:

[1] Zhu FC, Sun J, Yan GY, Huang JM, Chen C, He LS (2020). Insights into the strategy of micro-environmental adaptation: Transcriptomic analysis of two alvinocaridid shrimps at a hydrothermal vent. *Plos One* **15**, e0227587.

[2] Kikuchi T, Ohta S (1995). Two caridean shrimps of the families Bresiliidae and Hippolytidae from a hydrothermal field on the Iheya Ridge, off the Ryukyu Islands, Japan. *Journal of Crustacean Biology* **15**, 771–785.

[3] Tokuda G, Yamada A, Nakano K, Arita N, Yamasaki, H (2006). Occurrence and recent long-distance dispersal of deep-sea hydrothermal vent shrimps. *Biology Letters* **2**, 257–260.

[4] Yahagi T, Watanabe H, Ishibashi JI, Kojima S (2015). Genetic population structure of four hydrothermal vent shrimp species (Alvinocarididae) in the Okinawa Trough, Northwest Pacific. *Marine Ecology Progress Series* **529**, 159–169.
[5] Zhao Y, Xu T, Law YS *et al*. (2020). Ecological characterization of cold-seep epifauna in the South China Sea. *Deep Sea Research Part I: Oceanographic Research Papers* **163**, 103361.
[6] Sha Z, Wang Y (2018). Phylogenetic position of Alvinocarididae (Crustacea: Decapoda: Caridea): New insights into the origin and evolutionary history of the hydrothermal vent alvinocarid shrimps. *Deep Sea Research Part I: Oceanographic Research Papers* **141**, 93–105.
[7] Wang YR, Sha ZL (2017). A new species of the genus *Alvinocaris* Williams and Chace, 1982 (Crustacea: Decapoda: Caridea: Alvinocarididae) from the Manus Basin hydrothermal vents, Southwest Pacific. *Zootaxa* **4226**, 126–136.
[8] Van Audenhaege L, Fariñas-Bermejo A, Schultz T, Van Dover, CL (2019). An environmental baseline for food webs at deep-sea hydrothermal vents in Manus Basin (Papua New Guinea). *Deep Sea Research Part I: Oceanographic Research Papers* **148**, 88–99.

Table S3 Genebank accession numbers for the mitochondrial *cox1* genes of *Alvinocaris longirostris* and *A. kexueae* generated in this study.

No.	Species	Specimen ID	GenBank Accession No.	Region	Site	Habitat	Coordinate	Depth (m)
1	*A. longirostris*	AL_HM_X5	PP838275	South China Sea	Haima seep	Seep	16°43.8’N, 110°28.5’E	1,300–1,400
2	*A. longirostris*	AL_HM_X7	PP838276	South China Sea	Haima seep	Seep	16°43.8’N, 110°28.5’E	1,300–1,400
3	*A. longirostris*	AL_HM_X8	PP838277	South China Sea	Haima seep	Seep	16°43.8’N, 110°28.5’E	1,300–1,400
4	*A. longirostris*	AL_HM_XP1	OR048141	South China Sea	Haima seep	Seep	16°43.8’N, 110°28.5’E	1,300–1,400
5	*A. longirostris*	AL_HM_XP2	PP838278	South China Sea	Haima seep	Seep	16°43.8’N, 110°28.5’E	1,300–1,400
6	*A. longirostris*	AL_HM_XP3	OR048142	South China Sea	Haima seep	Seep	16°43.8’N, 110°28.5’E	1,300–1,400
7	*A. longirostris*	AL_HM_XP4	PP838279	South China Sea	Haima seep	Seep	16°43.8’N, 110°28.5’E	1,300–1,400
8	*A. longirostris*	AL_HM_XP5	PP838280	South China Sea	Haima seep	Seep	16°43.8’N, 110°28.5’E	1,300–1,400
9	*A. longirostris*	AL_HM_XP6	PP838281	South China Sea	Haima seep	Seep	16°43.8’N, 110°28.5’E	1,300–1,400
10	*A. longirostris*	AL_HM_XP8	PP838282	South China Sea	Haima seep	Seep	16°43.8’N, 110°28.5’E	1,300–1,400
11	*A. longirostris*	AL_HM_X10	PP838273	South China Sea	Haima seep	Seep	16°43.8’N, 110°28.5’E	1,300–1,400
12	*A. longirostris*	AL_HM_X11	PP838274	South China Sea	Haima seep	Seep	16°43.8’N, 110°28.5’E	1,300–1,400
13	*A. longirostris*	AL_HM_X12	PP838455	South China Sea	Haima seep	Seep	16°43.8’N, 110°28.5’E	1,300–1,400
14	*A. longirostris*	AL_HM_XR15	PP838283	South China Sea	Haima seep	Seep	16°43.8’N, 110°28.5’E	1,300–1,400
15	*A. longirostris*	AL_HM_XR17	PP838284	South China Sea	Haima seep	Seep	16°43.8’N, 110°28.5’E	1,300–1,400
16	*A. longirostris*	AL_HMROV2-1	PP838456	South China Sea	Haima seep	Seep	16°43.8’N, 110°28.5’E	1,300–1,400
17	*A. longirostris*	AL_HMROV1-0	OR048144	South China Sea	Haima seep	Seep	16°43.8’N, 110°28.5’E	1,300–1,400
18	*A. longirostris*	AL_HMROV1-2	PP838285	South China Sea	Haima seep	Seep	16°43.8’N, 110°28.5’E	1,300–1,400
19	*A. longirostris*	AL_HMROV1-3	PP838286	South China Sea	Haima seep	Seep	16°43.8’N, 110°28.5’E	1,300–1,400
20	*A. longirostris*	AL_HMROV2-2	PP838287	South China Sea	Haima seep	Seep	16°43.8’N, 110°28.5’E	1,300–1,400
21	*A. longirostris*	AL_SF_1	PP838288	South China Sea	Site F	Seep	22°07.0’N, 119°17.0’E	1,120–1,155
22	*A. longirostris*	AL_SF_2	PP838289	South China Sea	Site F	Seep	22°07.0’N, 119°17.0’E	1,120–1,155
23	*A. longirostris*	AL_SF_4	OR048150	South China Sea	Site F	Seep	22°07.0’N, 119°17.0’E	1,120–1,155
24	*A. longirostris*	AL_SF_5	PP838290	South China Sea	Site F	Seep	22°07.0’N, 119°17.0’E	1,120–1,155
25	*A. longirostris*	AL_SF_6	OR048151	South China Sea	Site F	Seep	22°07.0’N, 119°17.0’E	1,120–1,155
26	*A. longirostris*	AL_SF_8	OR048152	South China Sea	Site F	Seep	22°07.0’N, 119°17.0’E	1,120–1,155
27	*A. longirostris*	AL_SF_9	OR341155	South China Sea	Site F	Seep	22°07.0’N, 119°17.0’E	1,120–1,155

28	*A. longirostris*	AL_SF_10	PP838457	South China Sea	Site F	Seep	22°07.0’N, 119°17.0’E	1,120–1,155
29	*A. longirostris*	AL_SF_C21	PP838291	South China Sea	Site F	Seep	22°07.0’N, 119°17.0’E	1,120–1,155
30	*A. longirostris*	AL_SF_C3	PP838292	South China Sea	Site F	Seep	22°07.0’N, 119°17.0’E	1,120–1,155
31	*A. longirostris*	AL_SF_C4	OR048153	South China Sea	Site F	Seep	22°07.0’N, 119°17.0’E	1,120–1,155
32	*A. longirostris*	AL_SF_S1	PP838293	South China Sea	Site F	Seep	22°07.0’N, 119°17.0’E	1,120–1,155
33	*A. longirostris*	AL_SF_S2	PP838294	South China Sea	Site F	Seep	22°07.0’N, 119°17.0’E	1,120–1,155
34	*A. longirostris*	AL_SF_S3	PP838295	South China Sea	Site F	Seep	22°07.0’N, 119°17.0’E	1,120–1,155
35	*A. longirostris*	AL_SF_S4	PP838296	South China Sea	Site F	Seep	22°07.0’N, 119°17.0’E	1,120–1,155
36	*A. longirostris*	AL_SF_S5	PP838297	South China Sea	Site F	Seep	22°07.0’N, 119°17.0’E	1,120–1,155
37	*A. longirostris*	AL_SF_S6	PP838298	South China Sea	Site F	Seep	22°07.0’N, 119°17.0’E	1,120–1,155
38	*A. longirostris*	AL_SF_S7	PP838299	South China Sea	Site F	Seep	22°07.0’N, 119°17.0’E	1,120–1,155
39	*A. longirostris*	AL_MSA_A4268	OR048145	Manus Basin	Solwara 6	Vent	3°43.667’S, 151°40.869’E	1,708
40	*A. longirostris*	AL_MSA_A4269	PP838258	Manus Basin	Solwara 6	Vent	3°43.667’S, 151°40.869’E	1,708
41	*A. longirostris*	AL_MSA_A4271	PP838259	Manus Basin	Solwara 6	Vent	3°43.667’S, 151°40.869’E	1,708
42	*A. longirostris*	AL_MSA_A4273	PP838260	Manus Basin	Solwara 6	Vent	3°43.667’S, 151°40.869’E	1,708
43	*A. longirostris*	AL_MSA_A4274	OR048146	Manus Basin	Solwara 6	Vent	3°43.667’S, 151°40.869’E	1,708
44	*A. longirostris*	AL_MSA_A4275	OR048147	Manus Basin	Solwara 6	Vent	3°43.667’S, 151°40.869’E	1,708
45	*A. longirostris*	AL_MSA_A4276	PP838261	Manus Basin	Solwara 6	Vent	3°43.667’S, 151°40.869’E	1,708
46	*A. longirostris*	AL_MSA_A4304	OR048148	Manus Basin	Solwara 6	Vent	3°43.667’S, 151°40.869’E	1,708
47	*A. longirostris*	AL_MSA_A4948	PP838272	Manus Basin	South Su	Vent	3°43.667’S, 151°40.869’E	1,708
48	*A. kexueae*	AK_HM_1	OR048133	South China Sea	Haima seep	Seep	16°43.8’N, 110°28.5’E	1,300–1,400
49	*A. kexueae*	AK_HM_X4	OR048134	South China Sea	Haima seep	Seep	16°43.8’N, 110°28.5’E	1,300–1,400
50	*A. kexueae*	AK_HM_XP7	OR048135	South China Sea	Haima seep	Seep	16°43.8’N, 110°28.5’E	1,300–1,400
51	*A. kexueae*	AK_HM_XR13	OR048136	South China Sea	Haima seep	Seep	16°43.8’N, 110°28.5’E	1,300–1,400
52	*A. kexueae*	AK_HM_XR14	OR048137	South China Sea	Haima seep	Seep	16°43.8’N, 110°28.5’E	1,300–1,400
53	*A. kexueae*	AK_HM_XR16	OR048138	South China Sea	Haima seep	Seep	16°43.8’N, 110°28.5’E	1,300–1,400
54	*A. kexueae*	AK_HM_XS3	PP838216	South China Sea	Haima seep	Seep	16°43.8’N, 110°28.5’E	1,300–1,400
55	*A. kexueae*	AK_HMROV1	OR048139	South China Sea	Haima seep	Seep	16°43.8’N, 110°28.5’E	1,300–1,400
56	*A. kexueae*	A7735	PP838217	Manus Basin	South Su	Vent	3°48.404’S, 152°6.298’E	1,710

57	*A. kexueae*	A4947	OR048128	Manus Basin	South Su	Vent	3°48.404'S, 152°6.298'E	1,710
58	*A. kexueae*	A4949	OR048129	Manus Basin	South Su	Vent	3°48.404'S, 152°6.298'E	1,710
59	*A. kexueae*	A4950	OR048130	Manus Basin	South Su	Vent	3°48.404'S, 152°6.298'E	1,710
60	*A. kexueae*	A4951	PP838458	Manus Basin	South Su	Vent	3°48.404'S, 152°6.298'E	1,710
61	*A. kexueae*	A4952	PP838459	Manus Basin	South Su	Vent	3°48.404'S, 152°6.298'E	1,710
62	*A. kexueae*	A4954	OR048131	Manus Basin	South Su	Vent	3°48.404'S, 152°6.298'E	1,710
63	*A. kexueae*	A6946	OR341156	Manus Basin	South Su	Vent	3°48.404'S, 152°6.298'E	1,710
64	*A. kexueae*	A6957	OR048132	Manus Basin	South Su	Vent	3°48.404'S, 152°6.298'E	1,710

Table S4 Marginal likelihoods estimated based on different clock and prior tree models for the Bayesian skyline plot (BSP) analysis.

Sample	Clock model	Ln P (data|model)	Bayes Factor
A. longirostris (All individuals)	Strict clock	-1119.551	0
	**Relaxed Clock Exponential**	**-1126.942**	**7.391**
	Relaxed Clock Log-Normal	-1123.006	3.935
	Random Local Clock	-1121.712	2.162
A. kexueae (All individuals)	**Strict clock**	**-944.27**	**0**
	Relaxed Clock Exponential	-937.102	-7.168
	Relaxed Clock Log-Normal	-938.379	-5.891
	Random Local Clock	-939.13	-5.14
A. kexueae (Haima seep genetic group)	Strict clock	-834.24	0
	Relaxed Clock Exponential	-832.616	-1.624
	Relaxed Clock Log-Normal	-832.373	-1.867
	**Random Local Clock**	**-848.004**	**13.764**
A. kexueae (Manus Basin genetic group)	Strict clock	-885.081	0
	Relaxed Clock Exponential	-882.889	-2.916
	**Relaxed Clock Log-Normal**	**-885.805**	**0.44**
	Random Local Clock	-884.641	-1.752

Notes: The best model was indicated in bold.

Table S4 The bottom of pairwise Kimura 2-parameter (K2P) distance of [illegible] in this study

Region	Site	Specimen ID/ GenBank Accession	PP83827	PP83827	PP83845	PP83827	PP83827	PP83827	OR04814	PP83827	OR04814	PP83827	PP83828	PP83828	PP83828	PP83828	PP83828	PP83845	OR04814	PP83828	[illegible]
SCS	HM	PP838233																			
		PP838234	0.00000																		
		PP838455	0.00161	0.00161																	
		PP838235	0.00000	0.00000	0.00161																
		PP838236	0.00000	0.00000	0.00161	0.00000															
		PP838237	0.00000	0.00000	0.00161	0.00000	0.00000														
		OR048141	0.00161	0.00161	0.00323	0.00161	0.00161	0.00161													
		PP838278	0.00000	0.00000	0.00161	0.00000	0.00000	0.00000	0.00161												
		OR048142	0.00161	0.00161	0.00323	0.00161	0.00161	0.00161	0.00323	0.00161											
		PP838279	0.00161	0.00161	0.00323	0.00161	0.00161	0.00161	0.00323	0.00161	0.00323										
		PP838280	0.00161	0.00161	0.00323	0.00161	0.00161	0.00161	0.00323	0.00161	0.00323	0.00323									
		PP838281	0.00000	0.00000	0.00161	0.00000	0.00000	0.00000	0.00161	0.00000	0.00161	0.00161	0.00161								
		PP838282	0.00000	0.00000	0.00161	0.00000	0.00000	0.00000	0.00161	0.00000	0.00161	0.00161	0.00161	0.00000							
		PP838283	0.00000	0.00000	0.00161	0.00000	0.00000	0.00000	0.00161	0.00000	0.00161	0.00161	0.00161	0.00000	0.00000						
		PP838284	0.00000	0.00000	0.00161	0.00000	0.00000	0.00000	0.00161	0.00000	0.00161	0.00161	0.00161	0.00000	0.00000	0.00000					
		PP838456	0.00161	0.00161	0.00322	0.00161	0.00161	0.00161	0.00322	0.00161	0.00322	0.00322	0.00322	0.00161	0.00161	0.00161	0.00161				
		OR048144	0.00000	0.00000	0.00161	0.00000	0.00000	0.00000	0.00161	0.00000	0.00161	0.00161	0.00161	0.00000	0.00000	0.00000	0.00000	0.00161			
		PP838285	0.00000	0.00000	0.00161	0.00000	0.00000	0.00000	0.00161	0.00000	0.00161	0.00161	0.00161	0.00000	0.00000	0.00000	0.00000	0.00161	0.00000		
		PP838286	0.00000	0.00000	0.00161	0.00000	0.00000	0.00000	0.00161	0.00000	0.00161	0.00161	0.00161	0.00000	0.00000	0.00000	0.00000	0.00161	0.00000	0.00000	
		PP838287	0.00000	0.00000	0.00161	0.00000	0.00000	0.00000	0.00161	0.00000	0.00161	0.00161	0.00161	0.00000	0.00000	0.00000	0.00000	0.00161	0.00000	0.00000	[illegible]
	[illegible]	PP838288	0.00000	0.00000	0.00161	0.00000	0.00000	0.00000	0.00161	0.00000	0.00161	0.00161	0.00161	0.00000	0.00000	0.00000	0.00000	0.00161	0.00000	0.00000	[illegible]
		PP838457	0.00161	0.00161	0.00323	0.00161	0.00161	0.00161	0.00323	0.00161	0.00323	0.00323	0.00323	0.00161	0.00161	0.00161	0.00161	0.00322	0.00161	0.00161	[illegible]
		PP838289	0.00000	0.00000	0.00161	0.00000	0.00000	0.00000	0.00161	0.00000	0.00161	0.00161	0.00161	0.00000	0.00000	0.00000	0.00000	0.00161	0.00000	0.00000	[illegible]
		OR048150	0.00161	0.00161	0.00323	0.00161	0.00161	0.00161	0.00323	0.00161	0.00323	0.00323	0.00323	0.00161	0.00161	0.00161	0.00161	0.00322	0.00161	0.00161	[illegible]
		PP838290	0.00161	0.00161	0.00323	0.00161	0.00161	0.00161	0.00323	0.00161	0.00323	0.00323	0.00323	0.00161	0.00161	0.00161	0.00161	0.00322	0.00161	0.00161	[illegible]
		OR048151	0.00323	0.00323	0.00488	0.00323	0.00323	0.00323	0.00485	0.00323	0.00485	0.00485	0.00485	0.00323	0.00323	0.00323	0.00323	0.00484	0.00323	0.00323	[illegible]
		OR048152	0.00161	0.00161	0.00322	0.00161	0.00161	0.00161	0.00322	0.00161	0.00322	0.00322	0.00322	0.00161	0.00161	0.00161	0.00161	0.00322	0.00161	0.00161	[illegible]
[illegible]	[illegible]	[illegible]	[illegible]	[illegible]	[illegible]	[illegible]	[illegible]	[illegible]	[illegible]	[illegible]	[illegible]	[illegible]	[illegible]	[illegible]	[illegible]	[illegible]	[illegible]	[illegible]	[illegible]	[illegible]	[illegible]

Table S6 Migration rates between *Alvinocaris longirostris* populations estimated based on a fragment of 622-bp mitochondrial *cox1* gene using MIGRATE.

Migration rate		Recipent population							
		**MB**	**HM**	**SF**	**HK**	**TK**	**IK**	**IS**	**OH**
**Source population**	**MB**	--	23.781	23.462	21.834	22.791	20.611	24.057	18.950
	**HM**	22.431	--	23.384	20.694	22.866	19.101	22.562	16.424
	**SF**	24.602	23.972	--	24.696	23.312	21.234	25.941	11.280
	**HK**	15.797	15.643	16.385	--	15.428	14.327	16.220	12.869
	**TK**	17.475	18.365	17.175	17.102	--	14.645	17.615	21.200
	**IK**	4.468	5.057	4.725	5.042	4.727	--	5.185	3.384
	**IS**	23.303	22.712	23.098	22.269	21.476	19.088	--	17.475
	**OH**	0.232	0.258	0.244	0.244	0.253	0.236	0.246	--

Notes: MB, Manus Basin vents (sites 9 and 11 in Fig. 1d); HM, Haima seep; SF, Site F seep; HK, Hatoma Knoll vent; TK, Tarama Knoll vent; IK, Irabu Knoll vent; IS: Izena Hole and Sakai Field vents; OH, Off Hatsushima seep.

Table S7 The heatmap of pairwise Kimura 2-parameter (K2P) distance of *Alvinocaris kexueae* in this study.

Region	Site	GenBank Accession No.	SCS								MB																		
			HM								MB																		
			OR048133	OR048134	OR048135	OR048136	OR048137	OR048138	PP838216	OR048139	KX825833	KX825834	KX825835	MH398098	MH714459	MK671473	MK671475	MK671476	PP838217	OR048128	OR048129	OR048130	PP838458	PP838459	OR048131	OR341156	OR048132
SCS	HM	OR048133																									
		OR048134	0.00178																								
		OR048135	0.00356	0.00178																							
		OR048136	0.00535	0.00356	0.00535																						
		OR048137	0.00356	0.00178	0.00356	0.00535																					
		OR048138	0.00356	0.00178	0.00356	0.00535	0.00356																				
		PP838216	0.00535	0.00356	0.00178	0.00714	0.00535	0.00535																			
		OR048139	0.00356	0.00178	0.00000	0.00535	0.00356	0.00356	0.00178																		
MB	MB	KX825833	0.00714	0.00535	0.00714	0.00894	0.00714	0.00714	0.00535	0.00714																	
		KX825834	0.00535	0.00356	0.00535	0.00714	0.00535	0.00535	0.00356	0.00535	0.00178																
		KX825835	0.00535	0.00356	0.00535	0.00714	0.00535	0.00535	0.00356	0.00535	0.00178	0.00000															
		MH398098	0.00535	0.00356	0.00535	0.00714	0.00535	0.00535	0.00356	0.00535	0.00178	0.00000	0.00000														
		MH714459	0.00535	0.00356	0.00535	0.00714	0.00535	0.00535	0.00356	0.00535	0.00178	0.00000	0.00000	0.00000													
		MK671473	0.00535	0.00356	0.00535	0.00714	0.00535	0.00535	0.00356	0.00535	0.00178	0.00000	0.00000	0.00000	0.00000												
		MK671475	0.00714	0.00535	0.00714	0.00894	0.00714	0.00714	0.00535	0.00714	0.00000	0.00178	0.00178	0.00178	0.00178	0.00178											
		MK671476	0.00714	0.00535	0.00714	0.00894	0.00714	0.00714	0.00535	0.00714	0.00356	0.00178	0.00178	0.00178	0.00178	0.00178	0.00356										
		PP838217	0.00714	0.00535	0.00714	0.00894	0.00714	0.00714	0.00535	0.00714	0.00356	0.00178	0.00178	0.00178	0.00178	0.00178	0.00356	0.00356									
		OR048128	0.00714	0.00535	0.00714	0.00894	0.00714	0.00714	0.00535	0.00714	0.00356	0.00178	0.00178	0.00178	0.00178	0.00178	0.00356	0.00356	0.00356								
		OR048129	0.00714	0.00535	0.00714	0.00894	0.00714	0.00714	0.00535	0.00714	0.00000	0.00178	0.00178	0.00178	0.00178	0.00178	0.00000	0.00356	0.00356	0.00356							
		OR048130	0.00894	0.00714	0.00894	0.01075	0.00894	0.00894	0.00714	0.00894	0.00535	0.00356	0.00356	0.00356	0.00356	0.00356	0.00535	0.00535	0.00535	0.00535	0.00535						
		PP838458	0.00714	0.00535	0.00714	0.00535	0.00714	0.00714	0.00535	0.00714	0.00356	0.00178	0.00178	0.00178	0.00178	0.00178	0.00356	0.00356	0.00356	0.00356	0.00356	0.00535					
		PP838459	0.00535	0.00356	0.00178	0.00714	0.00535	0.00535	0.00356	0.00178	0.00894	0.00714	0.00714	0.00714	0.00714	0.00714	0.00894	0.00894	0.00894	0.00894	0.00894	0.00714	0.00894				
		OR048131	0.00535	0.00356	0.00535	0.00714	0.00535	0.00535	0.00356	0.00535	0.00178	0.00000	0.00000	0.00000	0.00000	0.00000	0.00178	0.00178	0.00178	0.00178	0.00178	0.00356	0.00178	0.00714			
		OR341156	0.01072	0.00892	0.01072	0.01253	0.01072	0.01072	0.00892	0.01072	0.00713	0.00534	0.00534	0.00534	0.00534	0.00534	0.00713	0.00356	0.00713	0.00713	0.00713	0.00892	0.00713	0.01253	0.00534		
		OR048132	0.00535	0.00356	0.00535	0.00714	0.00535	0.00535	0.00356	0.00535	0.00178	0.00000	0.00000	0.00000	0.00000	0.00000	0.00178	0.00178	0.00178	0.00178	0.00178	0.00356	0.00178	0.00714	0.00000	0.00534	

Table S8 Migration rates between *Alvinocaris kexueae* populations estimated based on a fragment of 564-bp mitochondrial *cox1* gene using MIGRATE.

Migration rate		Recipent population	
		MB	HM
Source population	MB	--	3.970
	HM	7.829	--

Notes: MB, Manus Basin vents gentic group (sites 10 and 11 in Fig. 1d); HM, Haima seep genetic group.
